# Supplementary material for: miR‐9 modulates and predicts the response to radiotherapy and EGFR inhibition in HNSCC
Source: EMBO Mol Med. 2021 Jun 1;13(7):e12872. doi: 10.15252/emmm.202012872 (PMC8261495; doi:10.15252/emmm.202012872)
Supplement: Supplementary file 2 — Appendix [file EMMM-13-e12872-s003.pdf]

Supplementary Appendix

miR-9 modulates and predicts the response to radiotherapy and EGFR-inhibition in HNSCC

Francesca Citron[1,2], Ilenia Segatto[1], Lorena Musco[1], Ilenia Pellarin[1], Gian Luca Rampioni Vinciguerra[1,3], Giovanni Franchin[4], Giuseppe Fanetti[4], Francesco Miccichè[5], Vittorio Giacomarra[6], Valentina Lupato[6], Andrea Favero[1], Isabella Concina[1], Sanjana Srinivasan[2], Michele Avanzo[7], Isabella Castiglioni[8], Luigi Barzan[6], Sandro Sulfaro[9], Gianluigi Petrone[5], Andrea Viale[2], Giulio F. Draetta[2], Andrea Vecchione[3], Barbara Belletti[1] and Gustavo Baldassarre[1].

Table of content:

Appendix Figure S1

Appendix Figure S2

Appendix Figure S3

Appendix Figure S4

Appendix Figure S5

Appendix Figure S6

Appendix Table S1

Appendix Table S2

Appendix Table S3

Appendix Table S4

Appendix Table S5

Appendix Table S6

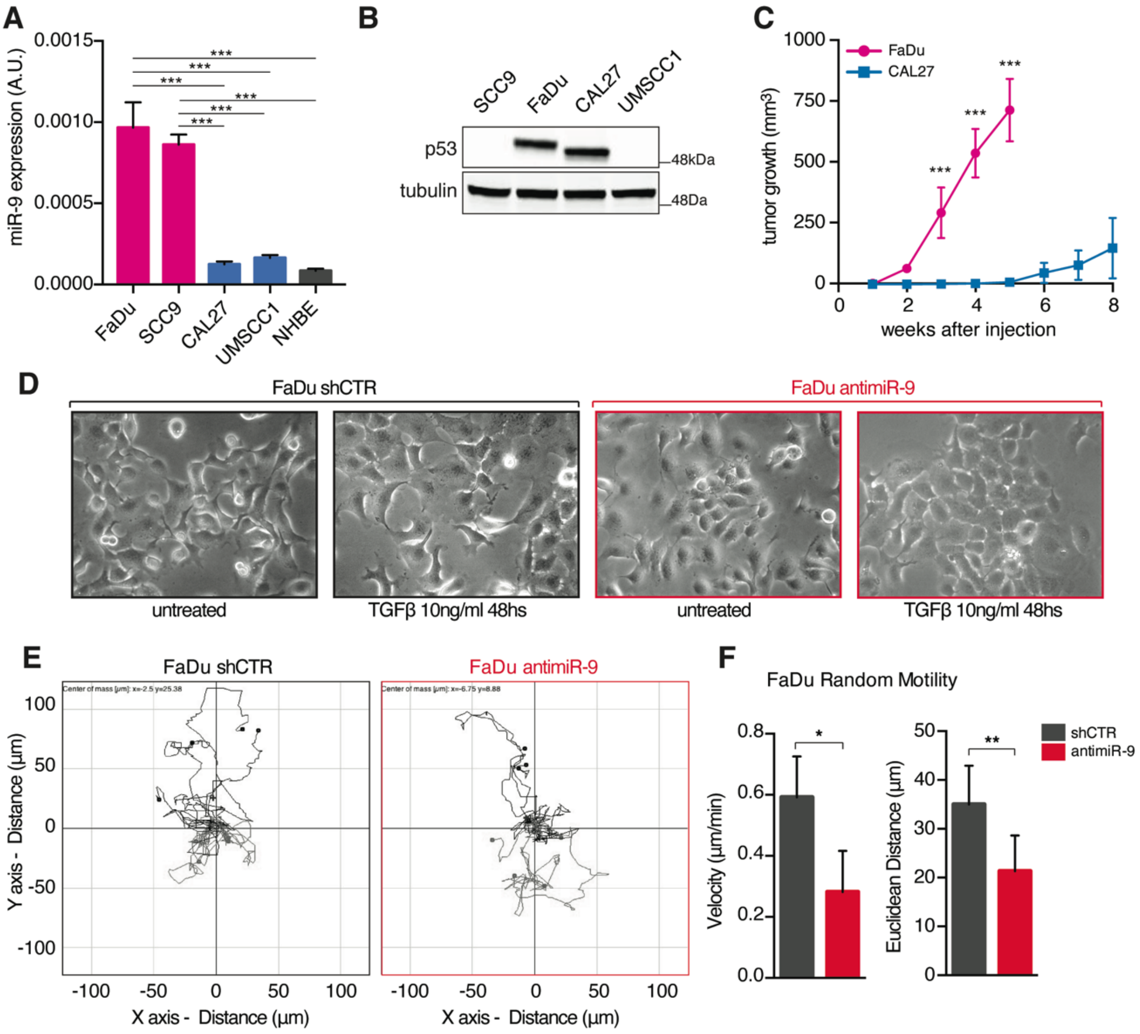

Appendix Figure S1. *miR-9 expression is associated with higher in vivo growth of HNSCC cells***.**

A. qRT-PCR analyses of normalized miR-9 expression in FaDu, SCC9, CAL27 and UMSCC1 cancer cells and NHBE normal epithelial cells. Data are the mean (±SD) of three independent experiments each performed in duplicate. A.U. = Arbitrary Uinits

B. Western Blot (WB) analyses of the p53 protein expression in FaDu, SCC9, CAL27 and UMSCC1 cells. Tubulin was used as loading control.

C. Graph reporting the mean value (±SD) of tumor volume in nude mice injected in both flanks with $1x10^6$ FaDu (n=4 mice) or $5x10^6$ CAL27 cells (n=3 mice) followed for up to 8 weeks.

D. Typical images of control (shCTR) and antimiR-9 FaDu cells not stimulated (untreated) or treated with TGFβ 10ng/mL for 48 hours.

E. Scatter plot reporting the distance covered by control (shCTR) and antimiR-9 FaDu cells as indicated in a typical analysis.

F. Graphs reporting the velocity and the distance covered by cells as in E. Data are the mean (±SD) of two independent experiments, performed in triplicate, in which at least 8 cells were tracked.

In A, C and F, unpaired t-test was used to verify the statistical significance. *p<0.05; **p<0.01; ***p<0.001

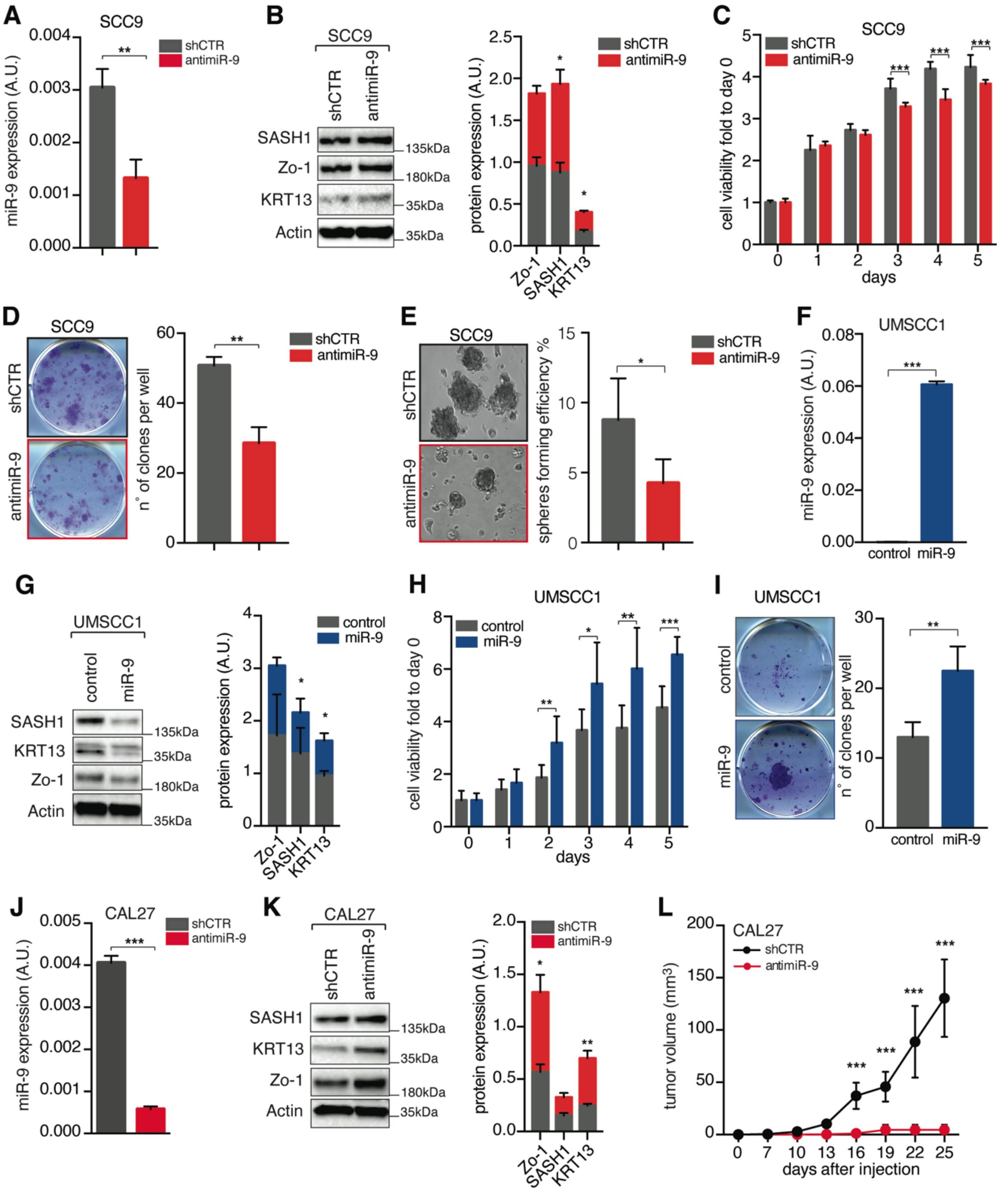

Appendix Figure S2. *MiR-9 expression sustains the tumor initiating properties of HNSCC cells*.

A. qRT-PCR analyses of normalized miR-9 expression in control (shCTR) and antimiR-9 SCC9 cells used in the experiment reported in B-E.

B. On the left, WB analyses of the indicated protein expression in control and antimiR-9 SCC9 cells described in B. Actin was used as loading control. Right graph shows the quantification of SASH1, KRT13 and Zo-1 protein expression. Data represent the mean (±SD) of three biological replicates.

C. Graph reporting the growth of SCC9 cells described in A-B followed up for 5 days using MTS assay and expressed as fold over day 1. Data represent the mean (±SD) of three independent experiments, performed in sextuplicate.

D. Colony formation assay of the cells described in A-B. On the left, representative images of the clones are shown, and on the right, the graph reports the number of clones per well.
E. Sphere forming assay with cells described in A-B. On the left, representative images of the spheres are shown, on the right, graph reports the percentage of sphere forming efficiency.
F. qRT-PCR analyses of normalized miR-9 expression in control and miR-9 overexpressing UMSCC1 cells used in the experiment reported in H-I.
G. On the left, WB analyses of the indicated protein expression in control and miR-9 overexpressing UMSCC1 cells described in F. Actin was used as loading control. Right graph shows the quantification of SASH1, KRT13 and Zo-1 protein expression. Data represent the mean (±SD) of three biological replicates.
H. Graph reporting the growth of UMSCC1 cells described in F-G followed up for 5 days using MTS assay and expressed as fold over day 1. Data represent the mean (±SD) of three independent experiments, performed in sextuplicate.
I. Colony formation assay of the cells described in F-G. On the left, representative images of the colonies are shown, and the graph on the right reports the number of colonies per well.
J. qRT-PCR analyses of normalized miR-9 expression in control (shCTR) and antimiR-9 CAL27 cells used in the experiment reported in K-L.
K. On the left, WB analyses of the indicated protein expression in control and antimiR-9 CAL27 cells described in J. Actin was used as loading control. Right graph shows the quantification of SASH1, KRT13 and Zo-1 protein expression. Data represent the mean (±SD) of three biological replicates.
L. Graph reporting tumor volume (mean ±SD) in NSG mice injected in both flanks with $1x10^6$ control (shCTR) or antimiR-9 CAL27 cells followed for up to 25 days (n=3 mice/group).
In the figure A.U. = Arbitrary Units; t-test was used to verify the statistical significance. *$p<0.05$; **$p<0.01$; ***$p<0.001$.

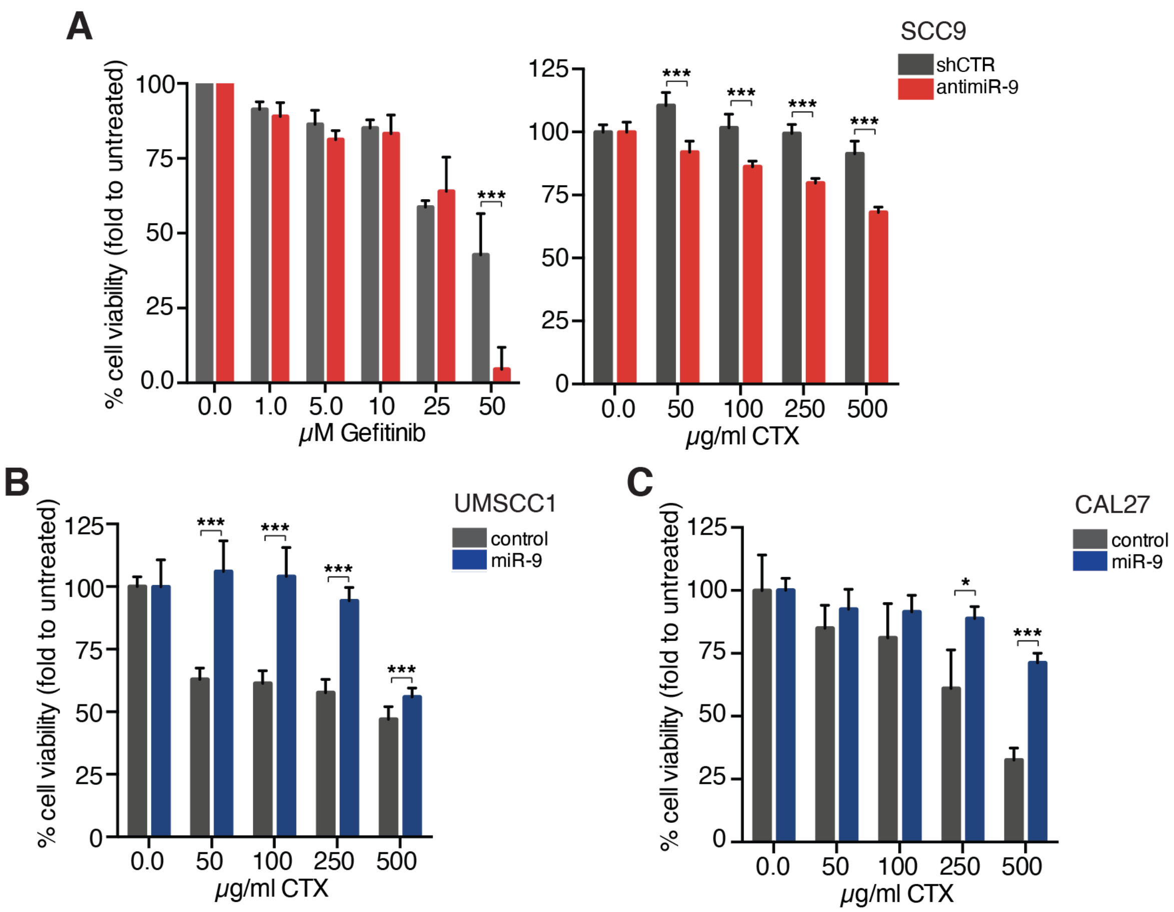

Appendix Figure S3. *miR-9 protects HNSCC cells from cell death induced by anti-EGFR therapies.*

A. Graphs report the cell viability of control (shCTR) and antimiR-9 SCC9 cells treated with increasing concentration of Gefitinib (left) or Cetuximab (CTX) for 72 hours and analyzed using MTS cell viability assay.

B/C. Graphs report cell viability of control and miR-9 UMSCC1 (B) or CAL27 (C) cells treated with increasing concentration of Cetuximab (CTX) for 72 hours and analyzed using MTS cell viability assay.

In the figure data represent the mean (±SD) of three independent experiments each performed in sextuplicate. Unpaired t-test was used to verify the statistical significance at each dose. *$p<0.05$; ***$p<0.001$.

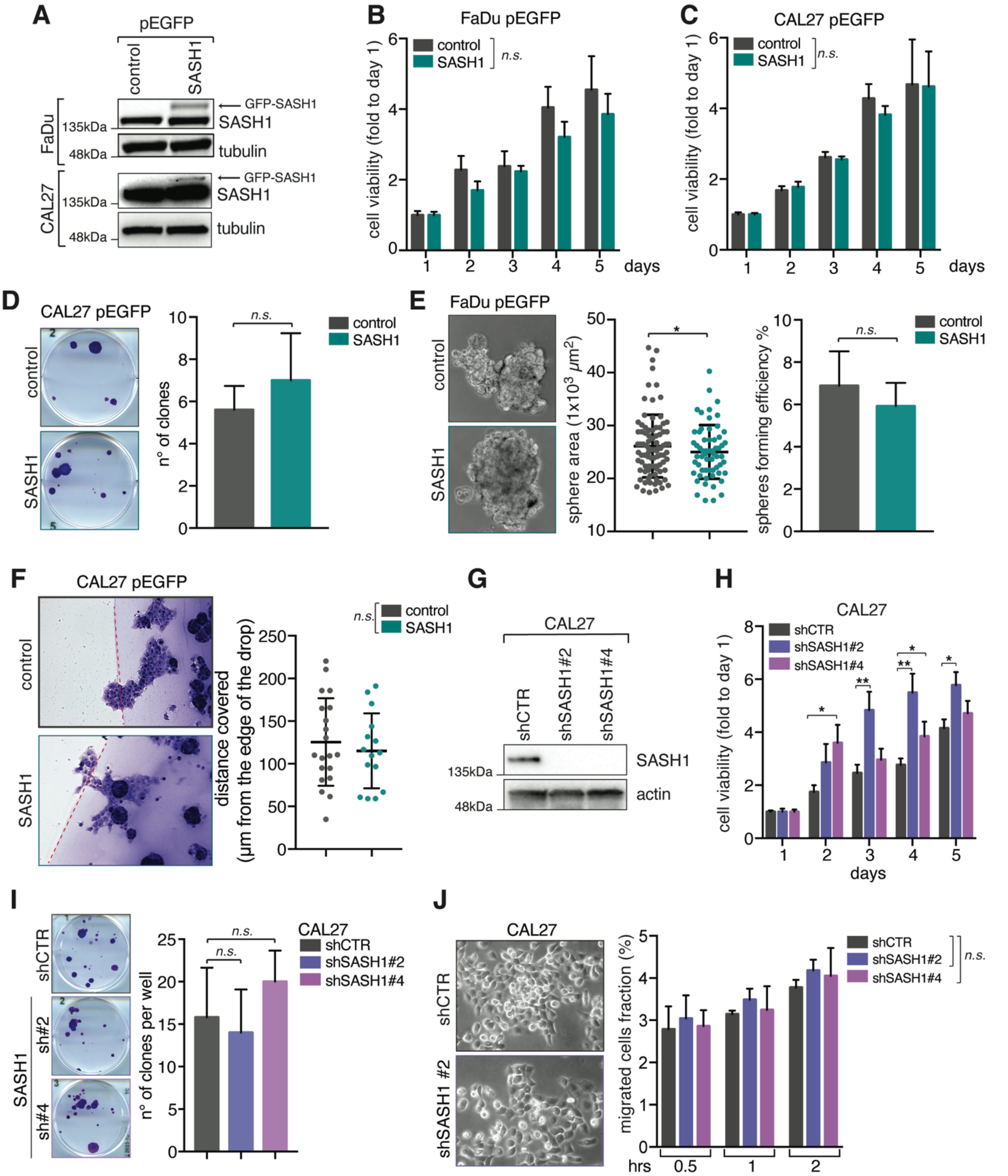

Appendix Figure S4. *SASH1 expression does not impact on proliferation, survival and motility of HNSCC cells.*

A. WB analyses of the indicated protein expression in control and SASH1 overexpressing FaDu (top panel) and CAL27 (bottom panel) cells. Tubulin was used as loading control.

B/C. Graph reporting the growth of control and SASH1 overexpressing FaDu (**B**) or CAL27 (**C**) cells followed up for 5 days using MTS assay. Data are expressed as fold over day 1, and represent the mean value (±SD) of three independent experiments, performed in sextuplicate.
D. Colony formation assay of the CAL27 cells described in A. On the left, representative images of the clones are shown, and on the right, graph reporting number of clones per well.
E. Sphere forming assay using FaDu cells described in A. On the left, representative images of the spheres are shown, on the right, graphs report the area of the sphere and percentage of sphere forming efficiency, as indicated.
F. Matrigel evasion assay of CAL27 cells described in A. On the right, representative phase-contrast images are reported. Left graph reports the distance covered by the individual cells from the edge of the drop.
G. WB analyses of SASH1 protein expression in control (shCTR) and SASH1 silenced (shSASH1#2 and #4) CAL27 cells. Actin was used as loading control.
H. Graph reporting the growth of CAL27 described in G and followed up for 5 days using MTS assay. Data are expressed as fold over day 1 and represent the mean (±SD) of three independent experiments, performed in sextuplicate. Two-way ANOVA with Sidak's multiple comparison test was used to verify the statistical significance.
I. Colony formation assay of the CAL27 cells described in G. On the left, representative images of the clones are shown, and on the right, graph reporting number of clones per well.
J. Cell migration assay of CAL27 cells described in G. On the left, representative images of CAL27 cells allowed to migrate for 2 hours in transwell assay and then allowed to attach for 8 hours. Right graph shows the percentage of migrated cells per well.
In the figure n.s = not significant; *$p<0.05$; **$p<0.01$.

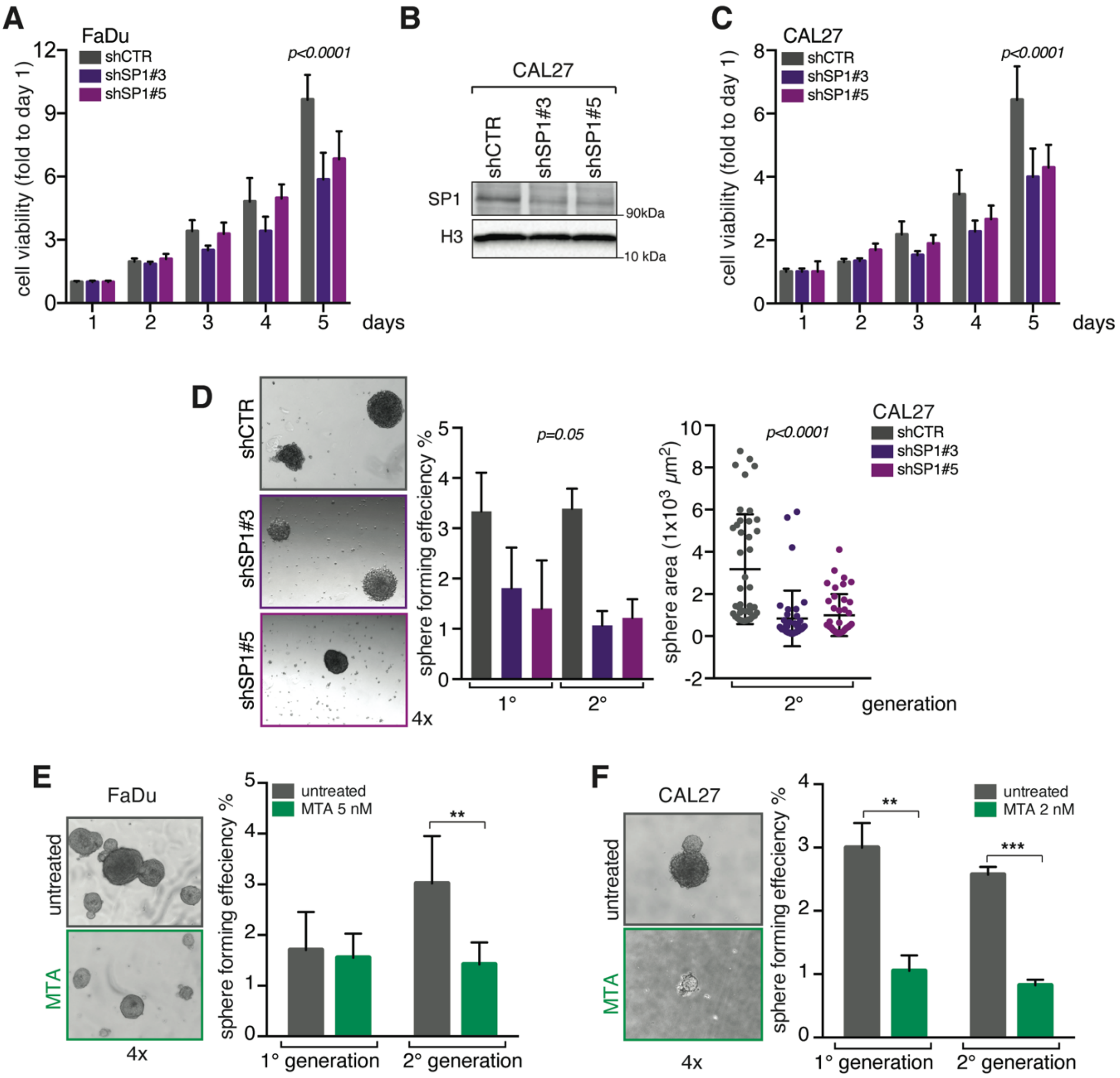

Appendix Figure S5. *Genetic or pharmacological inhibition of Sp1 phenocopies the miR-9 loss in HNSCC cells.*

A. Graph reporting the growth of FaDu cells silenced for Sp1 (shSP1#3 and #5) or not (shCTR) and followed up for 5 days using MTS assay. Data are expressed as fold over day 1 and represent the mean value (±SD) of three independent experiments, performed in sextuplicate. Two-way ANOVA with Sidak's multiple comparison test was used to verify the statistical significance.

B. WB analyses of Sp1 protein expression in control and Sp1 silenced CAL27 cells. Histone H3 was used as loading control.

C. Graph reporting the growth of CAL27 cells described in followed for 5 days using MTS assay. Data are expressed as fold over day 1 and represent the mean (±SD) of three independent experiments, performed in sextuplicate. Two-way ANOVA with Sidak's multiple comparison test was used to verify the statistical significance.

D. Sphere forming assay with cells described in B. On the left, representative images of the spheres are shown. The g/Fraphs report the percentage of sphere forming efficiency and the spheres area as indicated. Data are expressed as mean value (±SD) and two-way ANOVA with Sidak's multiple comparison test was used to verify the statistical significance.

E/F. Sphere forming assay with FaDu cells treated with 5 nMol/L (**E**) or CAL27 cells treated with 2 nMol/L of Mytramycin A (MTA) (**F**) or not (untreated). On the left, representative images of the spheres are shown. On the right, the graph reports the percentage of sphere forming efficiency in the first and second generation of spheres. Data are expressed as mean value (±SD) of three independent experiments performed in duplicate and two-way ANOVA test was used to calculate the statistical significance. **p<0.01; ***p<0.001.

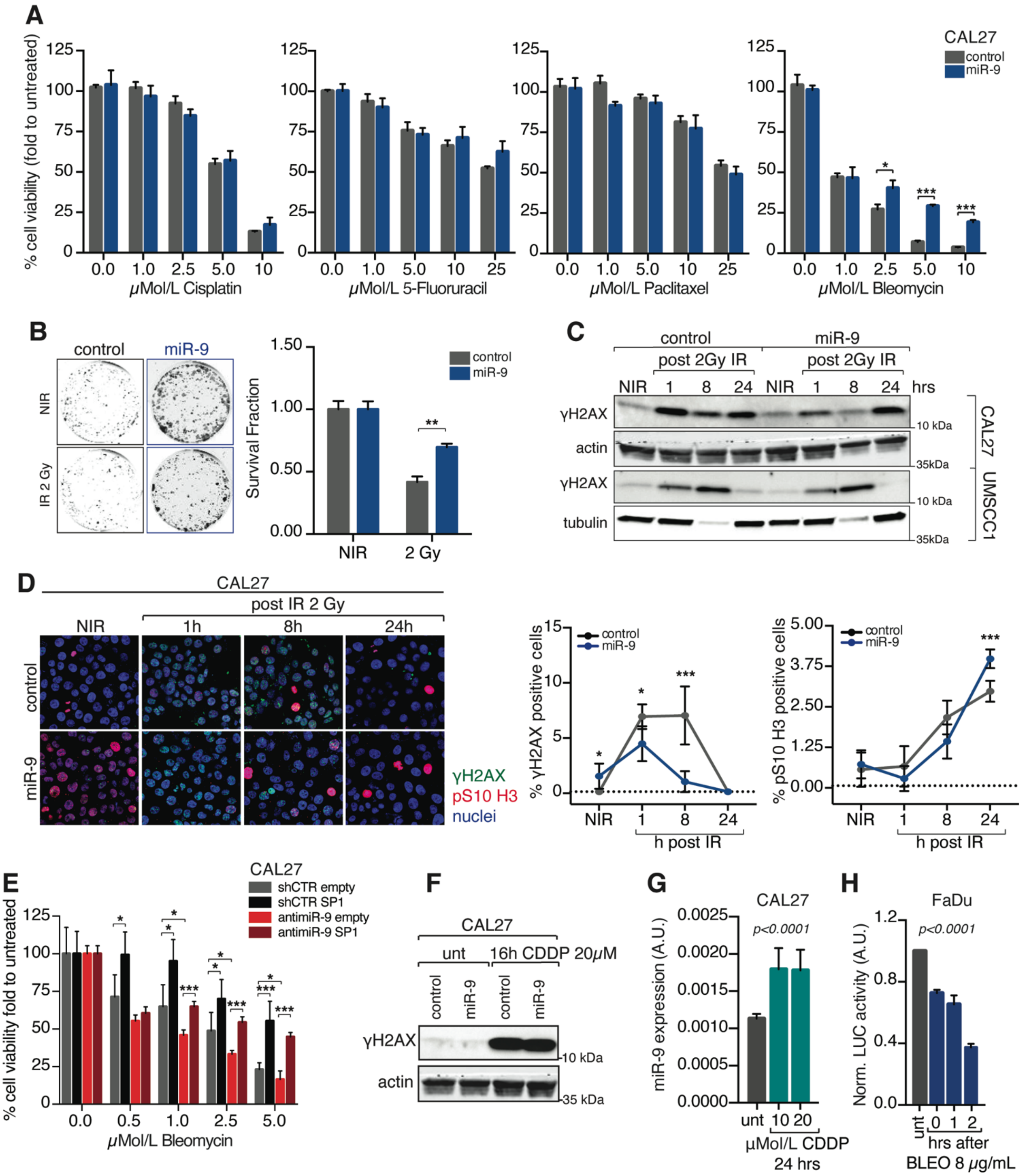

Appendix Figure S6. *Overexpression of miR-9 leads to radio-resistance in HNSCC cells.*

A. Graph reporting cell viability of control (shCTR) and miR-9 overexpressing CAL27 cells treated with increasing concentration of Cisplatin, 5-FluorUracil, Paclitaxel or Bleomycin for 72 hours and analyzed using MTS cell viability assay. Data represent the mean (±SD) of three independent experiments each performed in sextuplicate. Unpaired t-test was used to verify the statistical significance.

B. Clonogenic assay of CAL27 cells as described in A and treated with 2 Gy RT. The left panel shows typical images of the clones. Right graph reports the percentage (±SD) of survived cells respect to not irradiate cells (NIR). Unpaired t-test was used to verify the statistical significance.
C. WB analyses of the γH2AX expression in control (shCTR) and miR-9 overexpressing CAL27 (top panel) and UMSCC1 (bottom panel) cells not irradiated (NIR) and collected 1, 8 or 24 hours after 2Gy IR (F). Actin and tubulin were used as loading control.
D. Left panels show representative immunofluorescence staining of γH2AX (green), pS10-H3 (red) and nuclei (blue) performed in control (shCTR) and miR-9 overexpressing CAL27 cells, not irradiated (NIR) or irradiated with 2Gy, and collected at different time points (1h, 8h and 24 h post radiation). On the right, graphs reporting the percentage of γH2AX (left) and pS10-H3 (right) positive cells described in A evaluated in not irradiated (NIR) and 1, 8 or 24 hours after 2Gy IR using immunofluorescence analyses. Data represent the mean (±SD) of three independent experiments, in which at least 10 randomly selected fields were evaluated for the presence of γH2AX or pS10-H3 positive cells. Unpaired t-test was used to verify the statistical significance at each time point.
E. Graph reporting cell viability of control (shCTR) and antimiR-9 CAL27 cells, overexpressing or not SP1 and treated with increasing concentration of Bleomycin for 72 hours and analyzed using MTS cell viability assay. Data represent the mean (±SD) of three independent experiments each performed in sextuplicate. Two-way ANOVA with Sidak's multiple comparison test was used to verify the statistical significance.
F. WB analyses of the γH2AX expression in control and miR-9 overexpressing CAL27 cells treated with Cisplatin (CDDP) as indicated. Actin was used as loading control.
G. qRT-PCR analyses of normalized miR-9 expression in CAL27 cells treated with increasing concentration of CDDP, as indicated. Data represent the mean (±SD) of three independent experiments each performed in duplicate. One-way ANOVA test was used to verify the statistical significance.
H. Graph reporting the normalized Luciferase activity of miR-9 reporter vector in FaDu cells treated with Bleomycin (BLEO) for 16 hours (0) and then allowed to repair for 1 or 2 hours. Data represent the mean (±SD) of three independent experiments each performed in duplicate. One-way ANOVA test was used to verify the statistical significance.
In the figure *$p<0.05$; **$p<0.01$; ***$p<0.001$.

Head and Neck Cancer Samples	
**Characteristic**	**Value**
**Age** - Year	
Median	64
Range	33 -92
**Cancer site** - no. (%)	
Oral cavity / Tongue	58 (38.7)
Tongue	5 (3.3)
Oro-Pharynx	46 (30.7)
Hypo-Pharynx	11 (7.3)
Larynx	26 (17.3)
Tonsil	3 (2)
Nasal Cavity	1 (0.7)
**Tumor Grade -** no. (%)	
G1	10 (6.7)
G2	78 (52.0)
G3	53 (35.3)
Not Available or Specified	9 (6.0)
**Histology** - no. (%)	
SCC*	150 (100)
**cT**** - no. (%)	
T1	33 (22)
T2	56 (37.3)
T3	27 (18)
T4	29 (19.4)
Not Available or Specified	5 (3.3)
**cN***** - no. (%)	
N0	73 (48.67)
N1	28 (18.67)
N2	37 (24.67)
N3	2 (1.33)
Not Available or Specified	10 (6.67)

Appendix Table S1. *Patients and tumor features of the CRO-Aviano collection*

Appendix Table S1 summarizes the pathological and histological tumor status in HNSCC cohort collected at the CRO of Aviano. * SCC = Squamous Cells Carcinoma; ** cT = Clinical Tumor size *** cN = Clinical Node Status

sample ID	Hospital	Age	Sex	smoke	alcool	Histology	Site	Grading	HPV	TP53	cT	cN	cM	CT induction	Surgery	RT-Dose mGy	CTX	N° CYCLES	Response to treatment	Recurrence
**HN CET #2**	**CRO-Aviano**	81	M	N/A	N/A	SCC	LARYNX	N/A	N/A	N/A	3	0	0	NO	NO	7095	YES	8	CR	NO
**HN CET #3**	**CRO-Aviano**	80	M	EX	YES	SCC	LARYNX	G2	N/A	N/A	3	0	0	NO	NO	7740	YES	4	CR	NO
**HN CET #4**	**CRO-Aviano**	79	M	EX	YES	SCC	OROPHARINX	G3	NEG	MUT	2	2B	0	NO	NO	7095	YES	8	CR	NO
**HN CET #5**	**CRO-Aviano**	51	F	EX	NO	SCC	OROPHARINX	G3	N/A	N/A	N/A	N/A	N/A	SI	NO	6000	YES	4	PD	YES
**HN CET #7**	**CRO-Aviano**	77	M	EX	NO	SCC	OROPHARINX	G3	N/A	N/A	4	2b	0	NO	NO	6450	YES	3	PR	YES
**HN CET #8**	**CRO-Aviano**	77	M	EX	YES	SCC	OROPHARINX	G2	NEG	N/A	4b	2b	0	NO	NO	7000	YES	4	PD	YES
**HN CET #10**	**CRO-Aviano**	69	M	YES	YES	SCC	OROPHARINX	G2	NEG	N/A	4	2b	0	NO	NO	7000	YES	5	PR	NO
**HN CET #11**	**CRO-Aviano**	67	M	YES	YES	SCC	OROPHARINX	G3	NEG	N/A	3	3	0	NO	NO	7000	YES	2	PD	YES
**HN CET #12**	**CRO-Aviano**	71	M	EX	YES	SCC	LYMPHNODES	N/A	NEG	N/A	N/A	3	0	NO	NO	6450	YES	6	PR	YES
**HN CET #14**	**CRO-Aviano**	78	F	YES	EX	SCC	OROPHARINX	N/A	NEG	N/A	3	2c	0	NO	NO	7000	YES	3	PD	YES
**HN CET #15**	**CRO-Aviano**	52	M	SI	EX	SCC	IPOPHARYN	G3	N/A	N/A	1	2b	0	NO	NO	7095	YES	7	RC	NO
**HN CET #18**	**CRO-Aviano**	70	M	EX	YES	SCC	OROPHARINX	G3	N/A	N/A	1	2c	0	NO	NO	7095	YES	8	RC	NO
**HN CET #19**	**CRO-Aviano**	72	M	YES	EX	SCC	LARYNX	G3	POS	N/A	3	0	0	NO	NO	7095	YES	7	PR	YES
**HN CET #22**	**CRO-Aviano**	77	M	YES	YES	SCC	OROPHARINX	G3	NEG	MUT	3	2c	0	NO	NO	7095	YES	4	CR	NO
**HN CET #23**	**CRO-Aviano**	67	M	YES	YES	SCC	LARYNX	G2	N/A	N/A	3	0	0	YES	NO	7095	YES	5	PR	NO
**HN CET #25**	**CRO-Aviano**	44	M	EX	YES	SCC	OROPHARINX	G2	POS	N/A	3	2b	0	YES	NO	7095	YES	8	CR	NO
**HN CET #30**	**Gemelli**	65	F	N/A	N/A	SCC	OROPHARINX	G2	NEG	N/A	1	2c	0	NO	YES	7000	YES	7	PD	YES
**HN CET #31**	**Gemelli**	64	M	N/A	N/A	SCC	OROPHARINX	G2	N/A	N/A	3	2c	0	NO	YES	7680	YES	8	PR	YES
**HN CET #32**	**Gemelli**	62	M	NO	NO	SCC	OROPHARINX	G3	POS	N/A	4a	2b	0	NO	YES	7680	YES	4	PR	NO
**HN CET #33**	**Gemelli**	64	M	N/A	N/A	SCC	OROPHARINX	G2	N/A	N/A	3	2C	0	NO	YES	7680	YES	8	PR	YES
**HN CET #34**	**Gemelli**	54	M	YES	NO	SCC	OROPHARINX	G1	N/A	N/A	1	1	0	NO	YES	6600	YES	7	PR	NO
**HN CET #35**	**Gemelli**	65	M	N/A	N/A	SCC	OROPHARINX	N/A	N/A	N/A	4a	0	0	NO	NO	7020	YES	5	CR	NO
**HN CET #36**	**Gemelli**	56	F	N/A	N/A	SCC	OROPHARINX	G2	N/A	N/A	4a	2b	0	NO	NO	6600	YES	8	CR	NO
**HN CET #37**	**Gemelli**	54	F	NO	NO	SCC	OROPHARINX	N/A	N/A	N/A	4a	2b	0	NO	NO	6600	YES	4	CR	NO
**HN CET #38**	**Gemelli**	66	M	N/A	N/A	SCC	OROPHARINX	N/A	N/A	N/A	1	1	0	NO	YES	6600	YES	5	CR	NO
**HN CET #39**	**Gemelli**	71	M	YES	EX	SCC	OROPHARINX	G2	NEG	N/A	2	0	0	NO	NO	6600	YES	6	CR	NO
**HN CET #40**	**Gemelli**	55	M	N/A	N/A	SCC	OROPHARINX	N/A	POS	N/A	2	2c	0	NO	NO	6600	YES	5	CR	NO
**HN CET #41**	**Gemelli**	67	M	YES	NO	SCC	OROPHARINX	N/A	N/A	N/A	2	2b	0	NO	NO	6600	YES	5	PR	NO
**HN CET #42**	**Gemelli**	52	M	EX	NO	SCC	OROPHARINX	G2	N/A	N/A	4	3c	0	YES	YES	3060	YES	3	SD	YES
**HN CET #43**	**Gemelli**	56	F	N/A	N/A	SCC	OROPHARINX	G2	POS	N/A	2	2b	0	NO	NO	7000	YES	7	CR	NO
**HN CET #44**	**Gemelli**	56	F	YES	YES	SCC	OROPHARINX	N/A	NEG	N/A	3	1	0	NO	NO	6600	YES	6	CR	NO
**HN CET #45**	**Gemelli**	70	M	YES	YES	SCC	OROPHARINX	G2	N/A	N/A	1	0	0	NO	NO	6600	YES	6	CR	NO
**HN CET #46**	**Gemelli**	58	M	YES	EX	SCC	OROPHARINX	N/A	N/A	N/A	3	2c	0	NO	NO	6600	YES	6	CR	NO
**HN CET #48**	**Gemelli**	69	F	N/A	N/A	SCC	OROPHARINX	N/A	N/A	N/A	2	2b	0	NO	NO	6600	YES	5	PR	NO
**HN CET #49**	**Gemelli**	66	F	YES	NO	SCC	OROPHARINX	G3	N/A	N/A	1a	2b	0	NO	NO	6600	YES	5	CR	NO
**HN CET #50**	**Gemelli**	61	M	N/A	N/A	SCC	OROPHARINX	G2	POS	N/A	1	2b	0	NO	NO	7000	YES	4	CR	NO
**HN CET #51**	**Gemelli**	59	M	YES	NO	SCC	OROPHARINX	G2	N/A	N/A	2	2b	0	NO	NO	6600	YES	5	CR	NO

Appendix Table S2. *HNSCC patients treated with Radiotherapy and Cetuximab*

Appendix Table S2 summarizes the pathological and histological tumor status, including TP53 mutations and the presence of HPV infections in HNSCC cohort collected at CRO Aviano NCI and Gemelli Hospital.

Legend to Table S2

N/A	Not Available
SCC	Squamous Cells Carcinoma
cT	Clinical Tumor size
cN	Clinical Node Status
cM	Clinical Metastasis Status
CT	Chemotherapy
RT	Radiotherapy
CTX	Cetuximab
PD	Progression Disease
SD	Stable Disease
CR	Complete Response
PR	Partial Response

Primer	*Sequence 5'- 3'*	*GeneBank*
3'UTR KLF5 A For	TGGGCTCCCTCAAATGACAG	NM_001730
3'UTR KLF5 B Rev	GACCCCTTTTGGCATTTTGC	NM_001730
3'UTR KLF5 mut A	GGGAATACATTGTATTAATACCGGAGTGTTTG GTCATTTTAA	
3'UTR KLF5 mut B	GCTTATTTATTCTGCCCTCCGGTTAACAGCATC AGCATCAC	
Sp1 promoter -146 For	gctagcGGGCTTGTGGCGCGCTGCTC	NM_138473
Sp1 promoter -281 For	gctagcGCAACTTAGTCTCACACGCCTTGG	NM_138473
Sp1 promoter -443 For	gctagcCTATCAAAGCTTTGCCTATCC	NM_138473
Sp1 promoter -1612 For	gctagcGGCACCTAACACGGTAGGCAG	NM_138473
Sp1 promoter -20 Rev	ctcgagGCTCAAGGGGGTCCTGTCCGG	NM_138473

Appendix Table S3. *Primers used to clone Luciferase reporter vectors*.

Appendix Table S3 summarizes the sequences of the primers used to clone the different regions of the KLF5 3'-UTR and Sp1 promoter.

Gene	***Primer Forward 5' – 3'***	***Primer Reverse 5' – 3'***	***GeneBank ID***
ACTB	CCAGAGGCGTACAGGGATAG	CCAACCGCGAGAAGATGA	NM_001101
ALOXE3	CCCCATGAAAATTGACATCC	CATCCAGCTTCTTCCAGGAG	NM_021628
AMMECR1	GCAGAGATGTGCTGCTTTTG	AGCTCATCCCTTGTCATTGG	NM_015365
ATP10B	AGAAGCCAAAAAGGTGCTCA	ACAAGGTCTCCAACCACAGG	NM_025153
CD24	AACTAATGCCACCACCAAGG	CCTGTTTTTCCTTGCCACAT	NM_013230
EGFR	TGCGTCTCTTGCCGGAAT	GGCTCACCCTCCAGAAGCTT	NM_002211.3
ELOVL4	TTACACTGACTGCCCCTTCC	GCTCACACCATTTGCTGAAA	NM_005228
FAXDC2	ACAGCTTTCATCCTGGGCTC	AAAGTAGCCAGAAGCACCCC	NM_032385
FLG	GGCAAATCCTGAAGAATCCA	TGCTTTCTGTGCTTGTGTCC	NM_002016
JUP	GAAAAGCTGCTCTGGACCAC	GACGTTGACGTCATCCACAC	NM_001352773
KLF5	CCCTTGCACATACACAATGC	AGTTAACTGGCAGGGTGGTG	NM_001730
KLHL18	CTTGCTGCTGCAAAGAACAG	TGCACTGGCAAGAGAAAATG	NM_025010
LDLRAP1	CTTTCCTTCCTCCCCTTTTG	CTCAGGCACCTCCGTCTAAG	NM_015627
NCOA1	CATGGTCAGGCAAAAACCTT	GCTTGCCGATTTTGGTGTAT	NM_001362952
RASGRP1	GAGTTACATTGCCGCCTGAT	GTGAGGTGCTCGGATAGCTC	NM_005739
RCOR1	TTCAGGCAATCTCAGACGTG	CTTCGGGCATCTTAATGGAA	NM_015156
SASH1	CTGTCACCCCCTCAGTGTTT	GAACAGGGTGGAGTCCGTTA	NM_015278.3
SCYL3	TCAGTTGGTGTTTGCAGAGC	TTCAGCTGCTCCTGAGTGAA	NM_181093
SDHA	AGAAGCCCTTTGAGGAGCA	CGATTACGGGTCTATATTC	NM_004168.3
SERPINB8	CCACAATTCCAACACACAGC	TCCAGCCACACAGTGGAATA	NM_198833
SP1	GGTGCCTTTTCACAGGCTC	CATTGGGTGACTCAATTCTGCT	NM_138473

Appendix Table S4. *Primers used in qRT-PCR analyses***.**

Appendix Table S4 summarizes the sequences of the primers (forward and reverse) used for qRT-PCR analyses.

Antibody	Catalog Number	Vendor	Application and Dilution
SASH1	#A302-265A	Bethyl	WB (1:500)
pY1068-EGFR	#3777	Cell Signaling	WB (1:1000)
β-Actin	#8457	Cell Signaling	WB (1:2000)
pT202-Y204-ERK1/2	#9101	Cell Signaling	WB (1:1000)
ZO-1	#8193	Cell Signaling	WB (1:500)
Histone H3	#4499	Cell Signaling	WB (1:1000)
KRT13	#SAB2104755	Millipore-Sigma	WB (1:500)
α-Tubulin	#T8203	Millipore-Sigma	WB (1:2000)
Sp1	#SAB140397	Millipore-Sigma	WB (1:500)
p53	#OP43L	Millipore-Sigma	WB (1:1000)
pS139-H2AX (γH2AX)	#05-636	Millipore-Sigma	WB (1:1000), IF (1:500)
pS10-H3	#06-570	Millipore-Sigma	WB (1:1000), IF (1:500)
KLF5	sc-398470	Santa Cruz	WB (1:500)
ERK1	sc-271269	Santa Cruz	WB (1:1000)
EGFR	sc-03	Santa Cruz	WB (1:1000)
Vinculin	sc-73614	Santa Cruz	WB (1:1000)
Ki67 clone 30-9	#70-4286	Ventana	IHC (1:500)
Sp1	ab227383	Abcam	IHC (1:300)

Appendix Table S5. Primary antibodies**.**

Appendix Table S5 summarizes the primary antibodies (catalog number and vendor) and the dilution used in the different experiments. ***WB: Western Blot, IF: Immunofluorescence, IHC: Immunohistochemistry.

Amplified Region	Forward (5' – 3')	Reverse (5' – 3')	Reference (PMID)
SP1 -253/+7	GCAAGCGAGTCTTGCCATTGG	CGCTCATGGTGGCAGCTGAGG	This Manuscript
SP1 -480/-230	ATATCCCGGATTCTGGTTGGC	ATCCAATGGCAAGACTCGCT	This Manuscript
SP1 -673/-486	GCCCTCAGTTAATTCGGCGT	GCAAAATCCTAGTGGGCGGA	This Manuscript
SP1 -891/-674	CGCTAAAGCGTCCCACCTAA	GAAACTTGGAGTGGCAGAGGA	This Manuscript
SP1-1602/-1402	CGGTAGGCAGTCAGCAATCA	CCGGCCTTAATAGCTTGTCA	This Manuscript
Neg Ctr 1	TGGTACAACCACAGCTCAGTG	AAGCTGGACATGGTTGTGTG	32805052
Neg Ctr 2	CATCGAATGGAAATGAAAGGAGTC	ACCATTGGATGATTGCAGTCAA	19593370
Neg Ctr 3	CCTATTGTGGGTGGGAACCA	TGGTTTGCTCAGCCTACTTTCTTA	20875108
EPPK1	TGGGGCCTGGTGGGGGGAAAG	GGCCGGCCCCCTCTGACTCA	33827480
LAMC2	TCCCTAGCTGCGTTCTTTGC	AAGGTTGCAGATGCCAGTGAC	20875108
SERPINE1	CCCAAGACTCTCACCATTGACTT	GCTATGCTGCAGATGACCACAG	20875108
EPHA2	AACAGTTAAGTGGTCAGCAGAAAGG	GTAGCATGGCAGGGCAGC	20875108
INPP4B	AGCCTGATGGCGACTTTTGT	CTCTCTGGCTGCGTGTGAAG	20875108
SOX17	ATTAACTTCGGGGGCTCATT	CGGGAGCAGTTTACTTCCTG	24770696

Appendix Table S6. *Primers used in ChIP analyses***.**

Appendix Table S6 summarizes the sequences of the primers (forward and reverse) used for the amplification of different regions of SP1 promoter in the ChIP experiments. Negative (Neg Ctr 1, Neg Ctr 2, Neg Ctr 3) and positive (promoter of EPPK1, LAMC2, SERPINE1, EPHA2, INPP4B) were included to assess the specificity of the anti-KLF5 antibody.
