# Supplementary material for: miR‐9 modulates and predicts the response to radiotherapy and EGFR inhibition in HNSCC
Source: EMBO Mol Med. 2021 Jun 1;13(7):e12872. doi: 10.15252/emmm.202012872 (PMC8261495; doi:10.15252/emmm.202012872)
Supplement: Supplementary file 3 — Expanded View Figures PDF [file EMMM-13-e12872-s002.pdf]

Expanded View Figures

Figure EV1. miR-9 overexpression increases the tumor-initiating properties of HNSCC cells.

A qRT–PCR analyses of normalized miR-9 expression in control and miR-9 overexpressing CAL27 cells. Data represent the mean (± SD) of three independent experiments performed in duplicate. Unpaired *t*-test was used to verify the statistical significance.

B On the left, Western blot (WB) analyses of the indicated protein expression in CAL27 cells described in (A). Histone H3 was used as loading control. Right graph shows the quantification of indicated protein expression normalized on H3 expression. Data represent the mean (± SD) of three biological replicates. Unpaired *t*-test was used to verify the statistical significance.

C Cell viability analyses of cells described in (A, B) over a period of 5 days using MTS cell viability assay. Data represent the mean (± SD) of three independent experiments performed in sextuplicate. Unpaired *t*-test was used to verify the statistical significance per each time point.

D Colony formation assay of the cells described in (A, B). Left graph reports the number of clones per well. Right, representative images of clones are shown. Data represent the mean (± SD) of three independent experiments performed in duplicate. Unpaired *t*-test was used to verify the statistical significance.

E Sphere-forming assay of cells described in (A, B). Left graph reports the sphere-forming efficiency. Middle graph reports the area of first-generation spheres. Each dot represents one analyzed sphere. On the right, typical images of 10× field are shown. Data represent the median (± SD) of three independent experiments performed in duplicate. Unpaired *t*-test was used to verify the statistical significance.

F Left graph reports the tumor volume (mean ± SD) in NSG mice injected with control and miR-9 overexpressing CAL27 cells followed for up to 35 days ($n = 5$ mice/group). On the right, typical images of explanted tumors formed by control and miR-9 CAL27 cells at necropsy. Unpaired *t*-test was used to verify the statistical significance at each time point.

G Graph reporting the tumor onset (mean ± SD) in NSG mice injected with different numbers of control (shCTR) and miR-9 overexpressing CAL27 cells, as described, and followed for up to 35 days ($n = 5$ mice/group). Each dot represents a tumor. Two-way ANOVA with Sidak's multiple comparison test was used to calculate statistical significance.

H Typical images of hematoxylin & eosin (H&E—upper left panel) and Ki67 expression (Ki67—bottom left panel) evaluated by immunohistochemistry (IHC) in tumors explanted from mice described in (G). Right graph reports the percentage of Ki67-positive cells (mean ± SD). Each dot represents a tumor, and unpaired *t*-test was used to verify the statistical significance.

Data information: In the figure, A.U. = arbitrary units; *$P < 0.05$; **$P < 0.01$; ***$P < 0.001$.
Source data are available online for this figure.

© 2021 The Authors

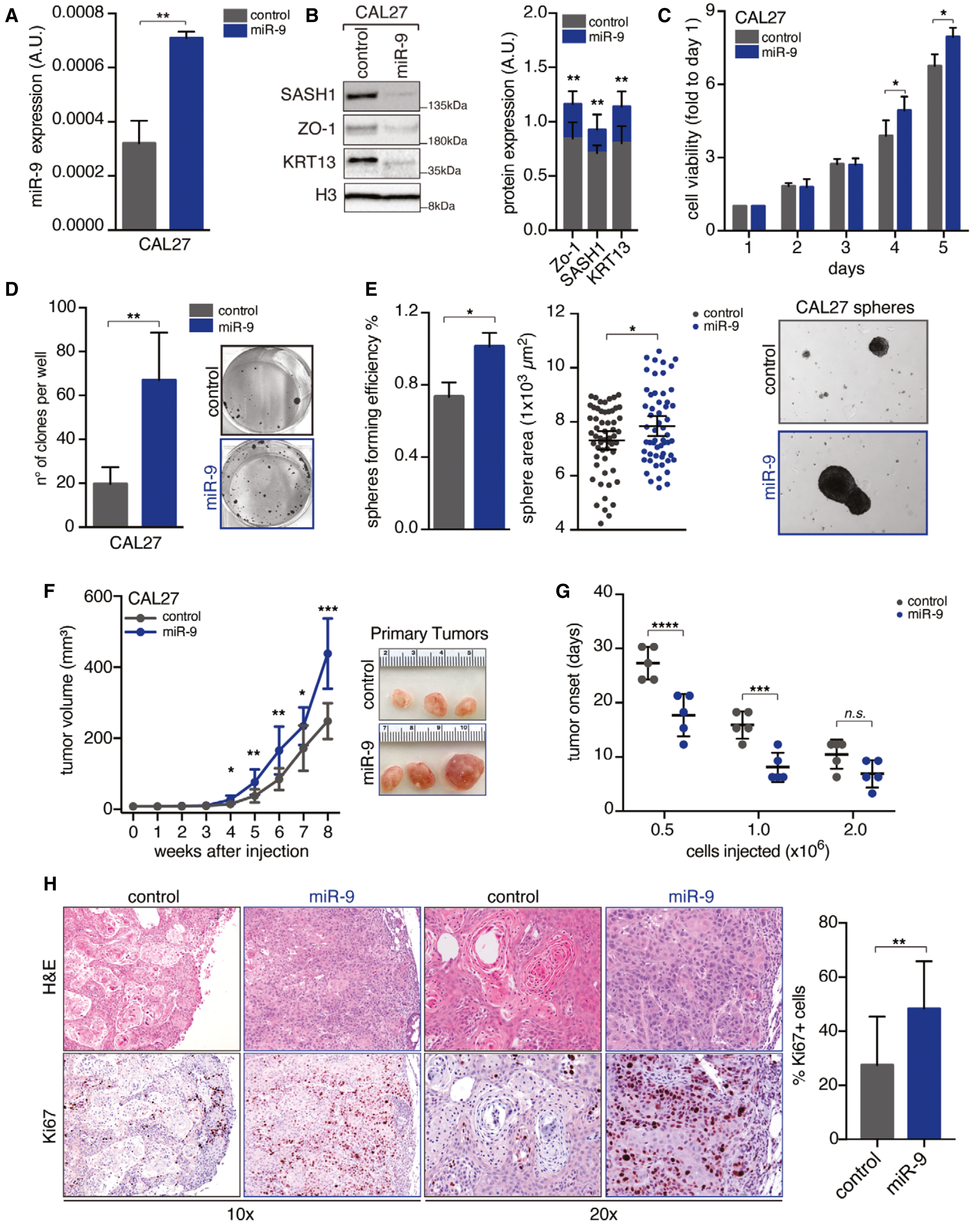

Figure EV1.

 © 2021 The Authors

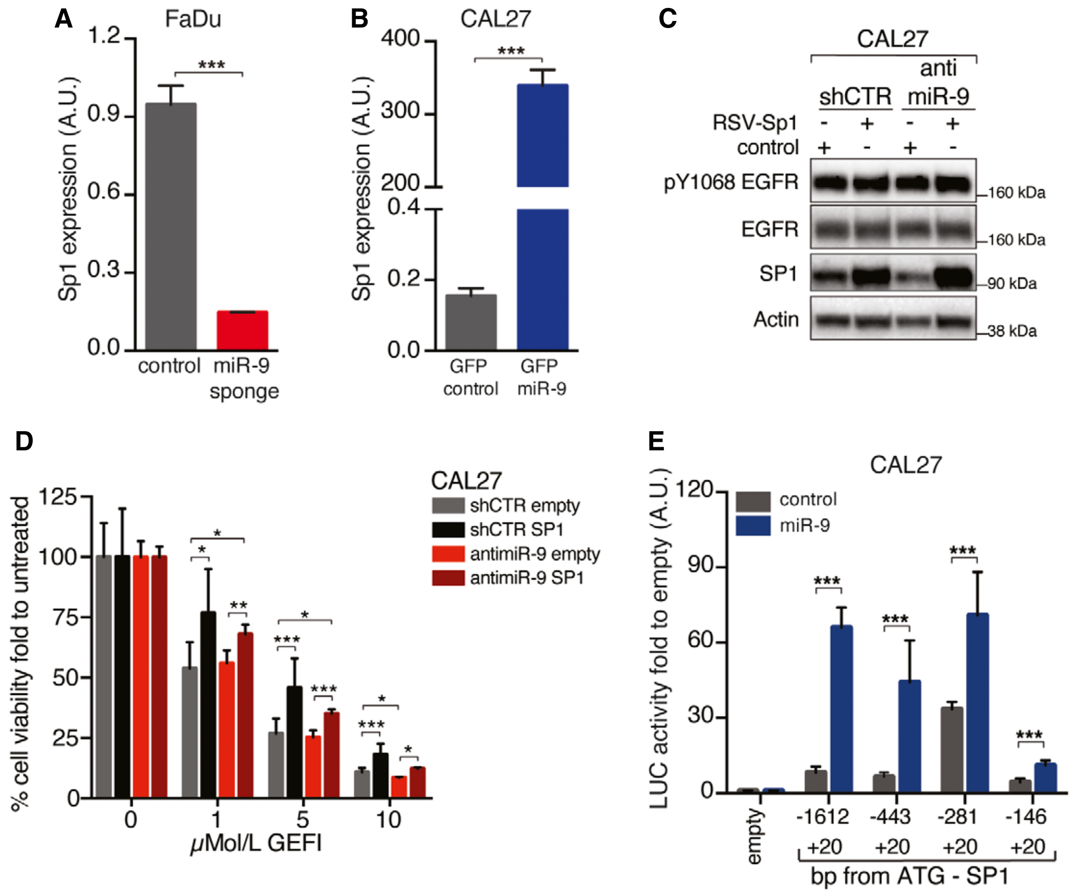

Figure EV2. miR-9 positively regulates Sp1 expression.

A qRT–PCR analyses of Sp1 expression in control (shCTR) and anti-miR-9 (miR-9 sponge) FaDu cells. Data represent the mean (± SD) of three independent experiments performed in duplicate, and unpaired *t*-test was used to verify the statistical significance.

B qRT–PCR analyses of Sp1 expression in CAL27 cells transiently transduced with and PGK-miR-9 (GFP-miR-9) or control (GFP control) vector as indicated. Data represent the mean (± SD) of three independent experiments performed in duplicate, and unpaired *t*-test was used to verify the statistical significance.

C WB analyses of the indicated protein expression in control (shCTR) and miR-9 CAL27 cells, overexpressing or not RSV-Sp1 as indicated. Actin was used as loading control.

D Graphs reporting the cell viability of CAL27 cells described in (C) and treated with increasing concentration of gefitinib (GEFI) and evaluated using the MTS assay. Data represent the mean (± SD) of two independent experiments performed in sextuplicate, and two-way ANOVA with Sidak's multiple comparison test was used to verify the statistical significance.

E Graph reporting the normalized luciferase activity of Sp1 promoter fragments in CAL27 cells control or stably overexpressing miR-9. Data are the mean (± SD) of three independent experiments performed in duplicate, and two-way ANOVA with Sidak's multiple comparison test was used to verify the statistical significance.

Data information: In the figure, A.U. = arbitrary units; $*P < 0.05$; $**P < 0.01$; $***P < 0.001$.
Source data are available online for this figure.

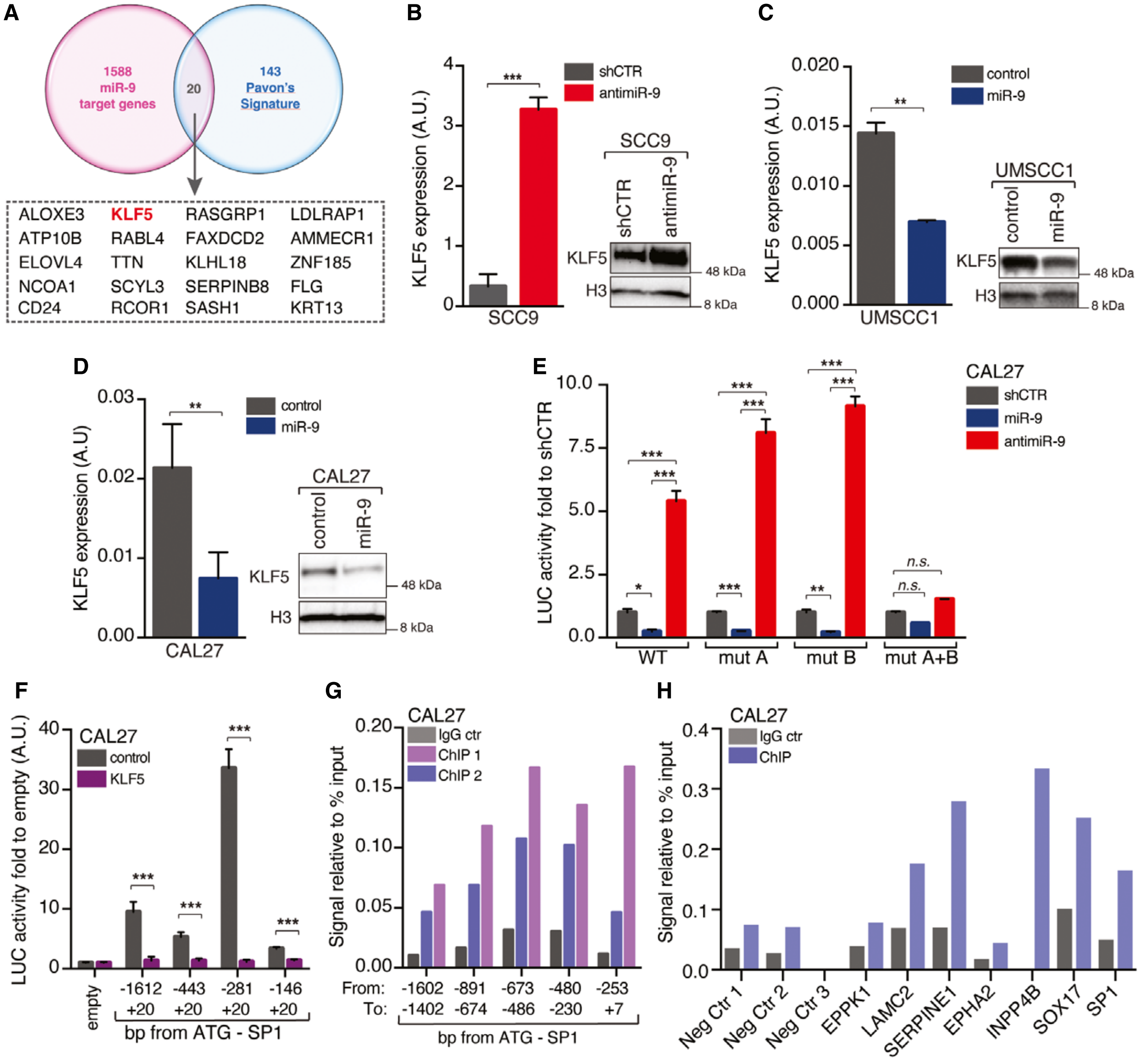

Figure EV3. miR-9 positively regulates Sp1 transcription by targeting KLF5.

A Venn Diagram showing the number of miR-9 potential targets among the genes altered during HNSCC progression as described in Pavón *et al* (2012).

B–D qRT–PCR WB analyses reporting KLF5 mRNA (left graph) and protein (right WB) expression in control (shCTR) or anti-miR-9 SCC9 cells (B), in control or miR-9 overexpressing UMSCC1 cells (C), or in control or miR-9 overexpressing CAL27 cells (D). Data represent the mean (± SD) of three independent experiments performed in duplicate, and unpaired *t*-test was used to verify the statistical significance. In WB analysis, histone H3 was used as loading control.

E Graph reporting the normalized luciferase activity of wild type (WT) or mutated KLF5 3′-UTR described in Fig 4C in control (shCTR), miR-9 overexpressing, or anti-miR-9 CAL27 cells, as indicated. Data represent the mean (± SD) of three independent experiments performed in duplicate, and two-way ANOVA with Sidak's multiple comparison test was used to verify the statistical significance.

F Graph reporting the normalized luciferase activity of Sp1 promoter fragments in CAL27 cells transfected with control or KLF5 vectors, as indicated. Data represent the mean (± SD) of three independent experiments performed in duplicate, and unpaired *t*-test was used to calculate the statistical significance.

G Chromatin immunoprecipitation (ChIP) assay performed on CAL27 cells. The graph reports the binding of KLF5 to the indicated region of Sp1 promoter expressed as signal relative to input in two independent immunoprecipitations (ChIP1 and ChIP2) using the KLF5 ab. IgG was used as negative control on the same chromatin.

H Chromatin immunoprecipitation (ChIP) assay performed on CAL27 cells. The graph reports the binding of KLF5 to the indicated genomic regions used as negative (Neg Ctr 1, Neg Ctr 2, and Neg Ctr 3) or potentially positive controls (EPPK1; LAM2C2; SERPINE1; EPHA2; INPP4B; SOX17) of anti-KLF5 antibody specificity. The Sp1 -673/-486 fragment was amplified on the same DNA recovered from the IP as internal reference.

Data information: In the figure, A.U. = arbitrary units; *$P < 0.05$; **$P < 0.01$; ***$P < 0.001$.
Source data are available online for this figure.

© 2021 The Authors

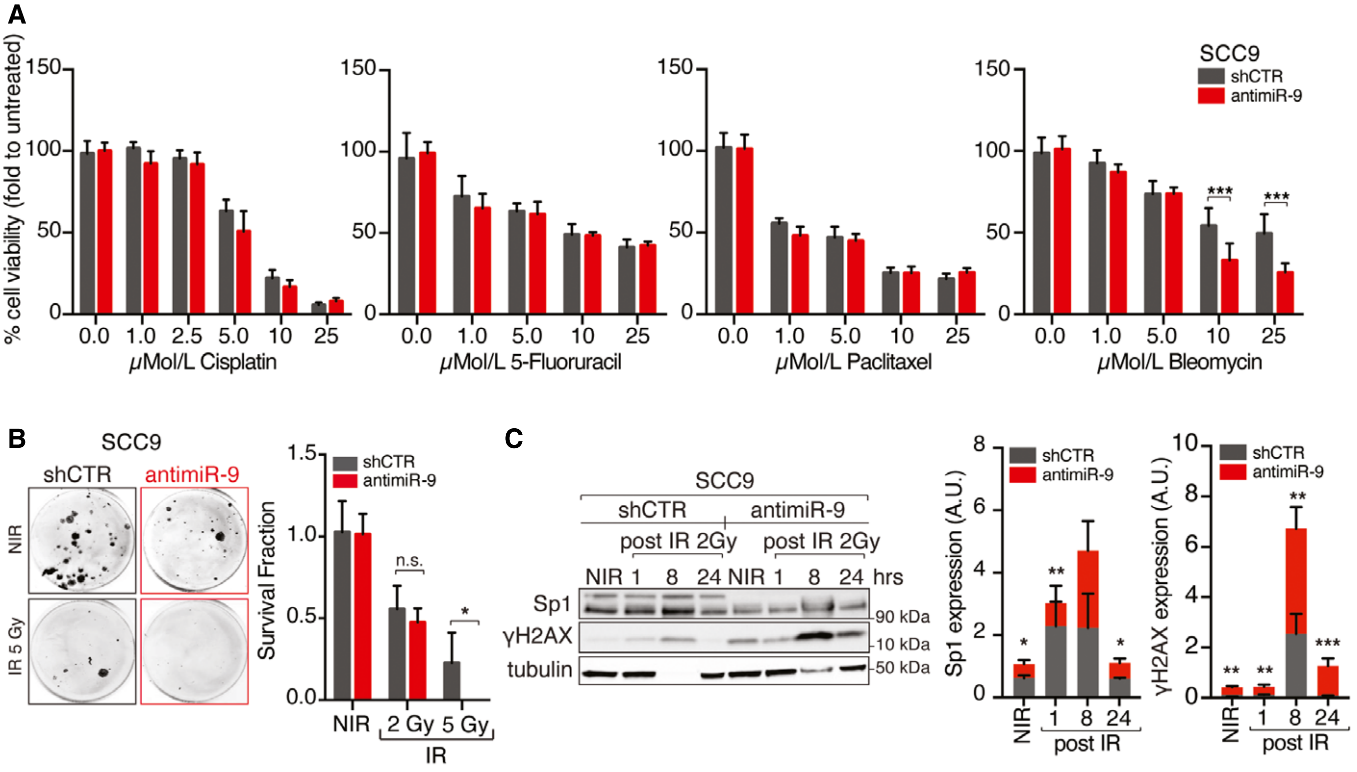

Figure EV4. miR-9 protects HNSCC cells from RT-induced cell death.

A Graph reporting cell viability of control (shCTR) and anti-miR-9 SCC9 cells treated with increasing concentration of the indicated drugs for 72 h and analyzed using the MTS cell viability assay. Data represent the mean (± SD) of three independent experiments each performed in sextuplicate. Unpaired *t*-test was used to calculate the statistical significance at each dose.

B Clonogenic assay of control (shCTR) and anti-miR-9 SCC9 cells not irradiated (NIR) or treated with 2 or 5 Gy IR. On the left, typical images of cell clones are shown, and on the right, the graph reports the percentage (± SD) of survived cells respect to not irradiate cells in three independent experiments performed in triplicate. Unpaired *t*-test was used to calculate the statistical significance at each dose.

C Left, WB analyses of the indicated protein expression in control (shCTR) and anti-miR-9 SCC9 cells not irradiated (NIR) or treated with 2 Gy IR and allowed to repair for the indicated hours (h). Tubulin was used as loading control. On the left, graphs report the quantification of the indicated proteins normalized on tubulin expression. Data are expressed as mean (± SD) of three independent experiments.

Data information: In the figure, A.U. = arbitrary units; unpaired *t*-test was used to calculate the statistical significance at time point.*$P < 0.05$; **$P < 0.01$; ***$P < 0.001$.
Source data are available online for this figure.

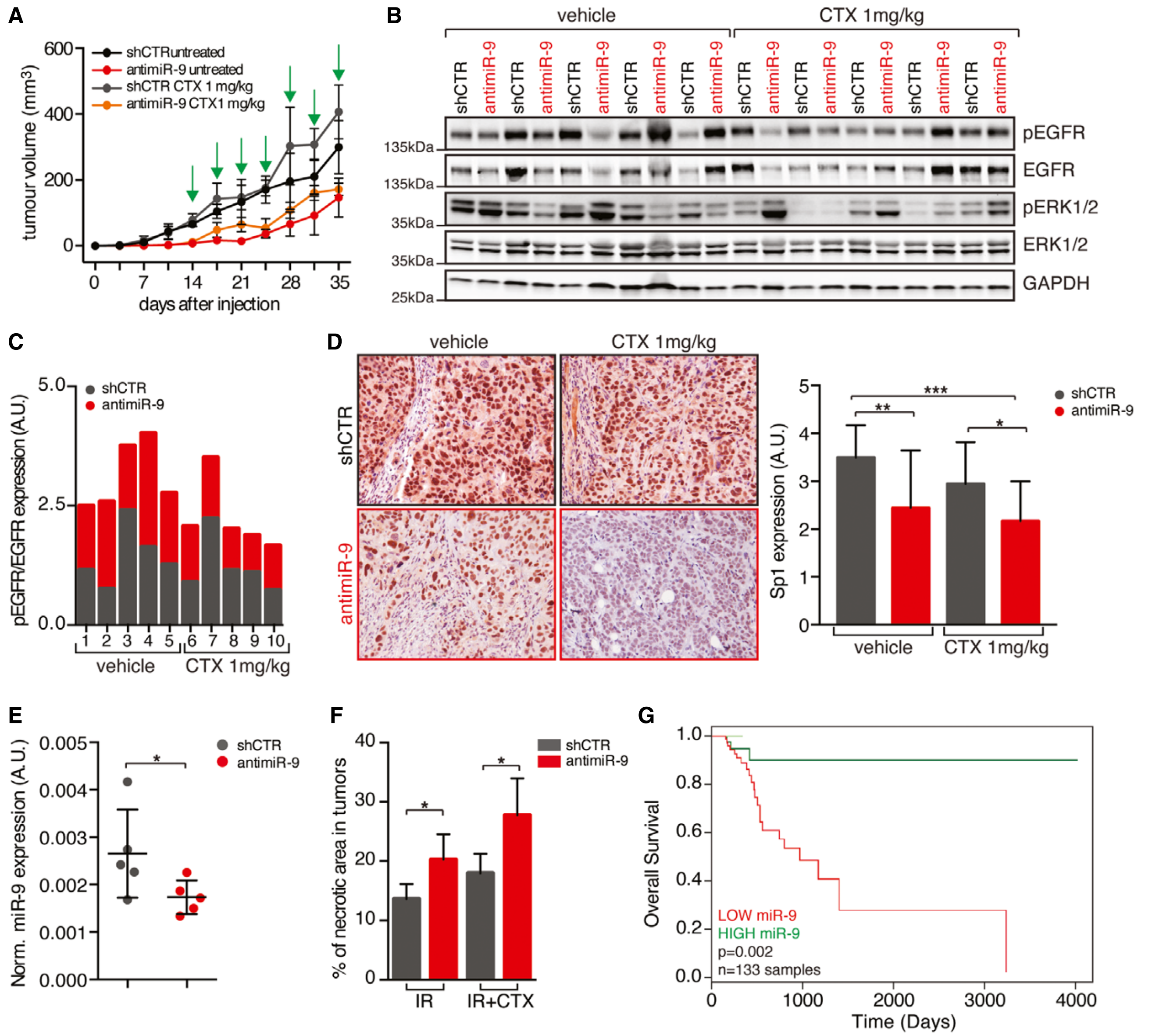

Figure EV5. miR-9 protects from RT+CTX-induced cell death, *in vivo*.

A Graph reporting the tumor volume of mice (n = 5 mice/group) injected in both flanks with control (shCTR) or anti-miR-9 FaDu cells and treated with vehicle (untreated) or Cetuximab (1 mg/kg, IP injections) every three days for 3 weeks. Data are represented as mean (± SD).

B WB analyses evaluating the expression of the indicated proteins in tumor explanted from mice described in (A). GAPDH was used as loading control.

C Graph reporting the normalized pY1068 EGFR expression folded over total EGFR in tumor explanted from mice described in (A).

D On the left, typical images of Sp1 expression evaluated by immunohistochemistry (IHC) in tumors (n = 5 mice/group) explanted from mice treated as in (A). On the right, graph reports the Sp1 expression scored from 0 (negative) to 4 (strong) based on its nuclear intensity by a blinded pathologist. Data are represented as mean (± SD), and two-way ANOVA with Sidak's multiple comparison test was used to verify the statistical significance.

E Dot plot reporting the qRT–PCR of normalized miR-9 expression in tumor treated with control (shCTR) and anti-miR-9 lentiviruses FaDu cells as described in Fig 6A. Each dot represents a tumor. Data are represented as mean (± SD), and two-way ANOVA test was used to calculate statistical significance.

F Graph reporting the percentage of necrotic areas in tumors injected with control and anti-miR-9 lentiviruses and treated with radiotherapy alone (IR) or in combination with Cetuximab (IR+CTX) as described in Fig 6A. Data are represented as mean (± SD), and two-way ANOVA with Sidak's multiple comparison test was used to verify the statistical significance.

G Kaplan–Mayer curve evaluating the overall survival of HNSCC patients treated with RT+Cisplatin combination included in the TCGA dataset, segregated on the expression of miR-9 in the primary tumor (high expression ≥ 75,819 reads n = 34; low expression < 75,819 reads n = 99). Number of total evaluated samples (n) and P value are reported in the graph. Statistical significance was calculated with log-rank test.

Data information: In the figure, A.U. = arbitrary units; *$P < 0.05$; **$P < 0.01$; ***$P < 0.001$.
Source data are available online for this figure.

 © 2021 The Authors
