# Supplementary figures and images for: miR‐9 modulates and predicts the response to radiotherapy and EGFR inhibition in HNSCC
Source: EMBO Mol Med. 2021 Jun 1;13(7):e12872. doi: 10.15252/emmm.202012872 (PMC8261495; doi:10.15252/emmm.202012872)

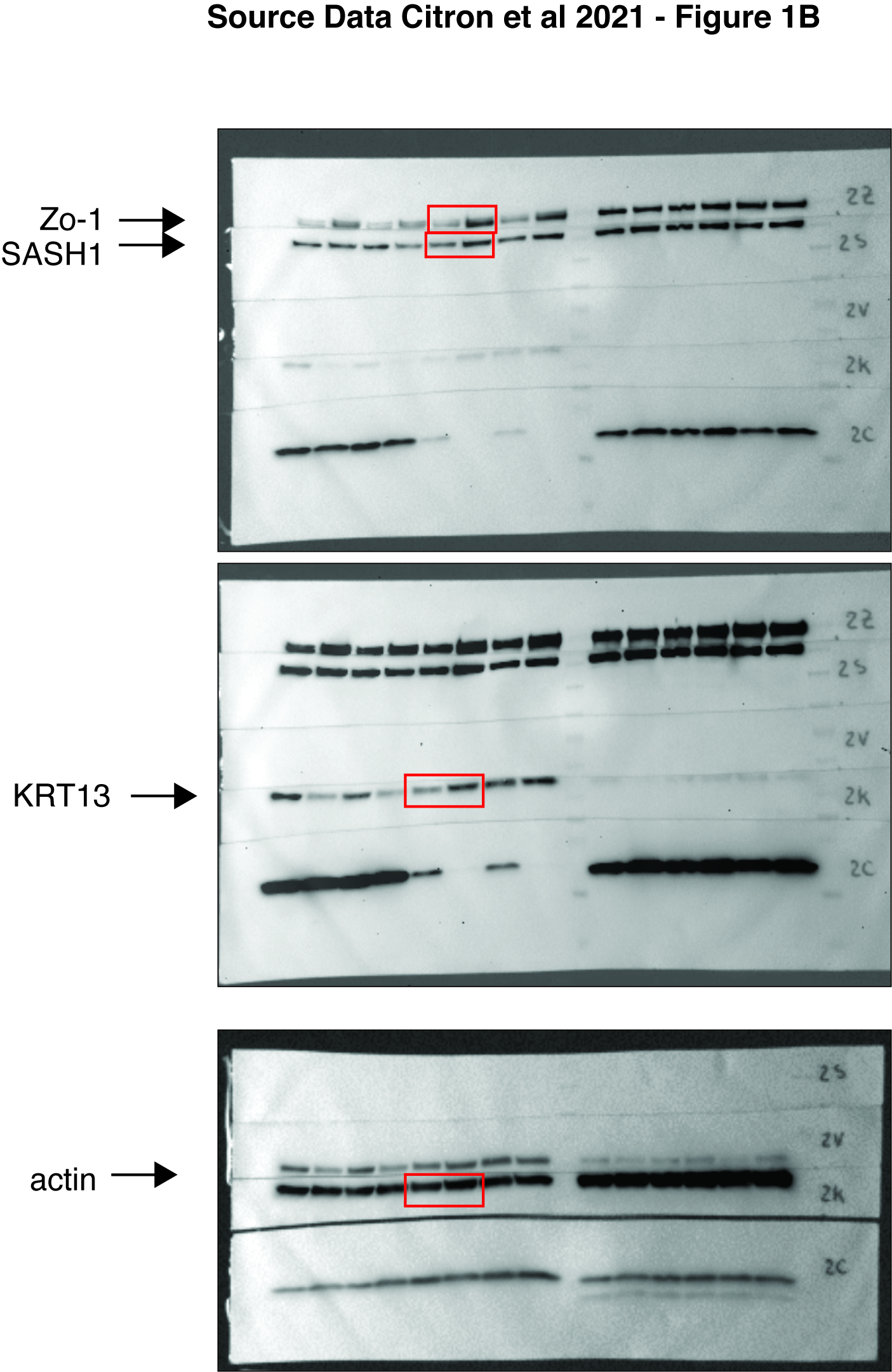

Supplement: Supplementary file 4 — Source Data for Figure 1 [file EMMM-13-e12872-s006.tif]

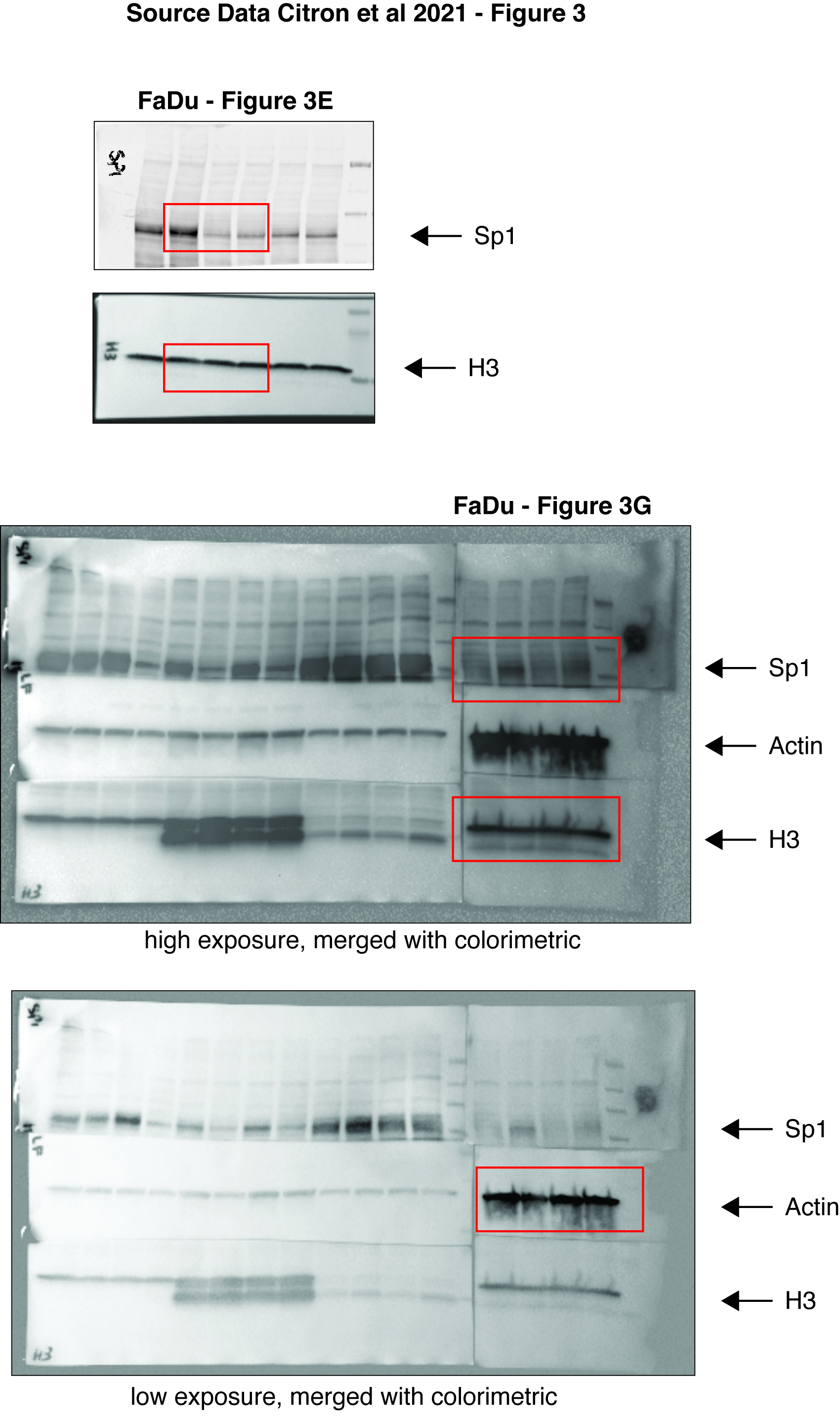

Supplement: Supplementary file 5 — Source Data for Figure 3 [file EMMM-13-e12872-s001.tif]

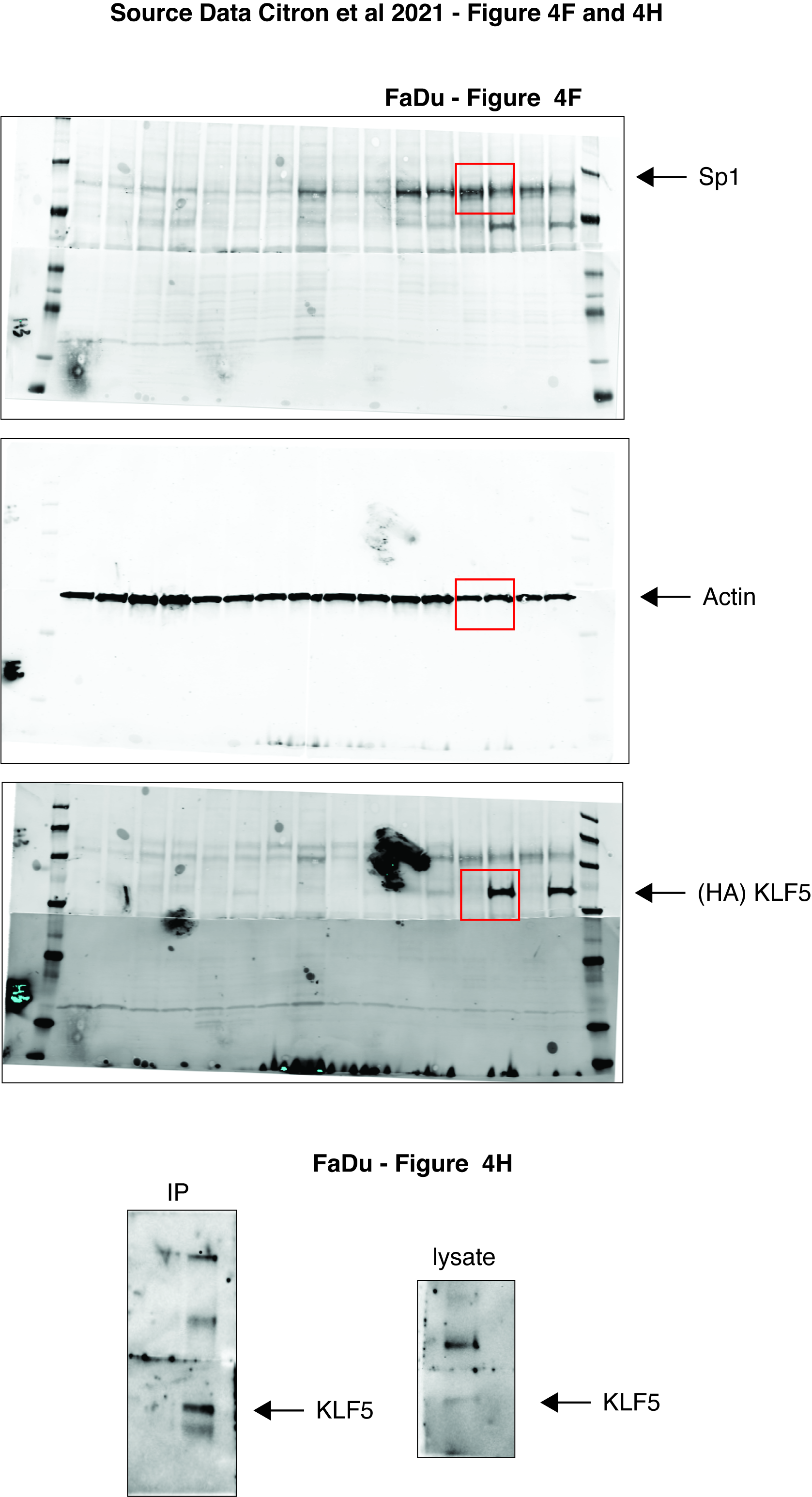

Supplement: Supplementary file 6 — Source Data for Figure 4 [file EMMM-13-e12872-s007.zip › Raw Data Fig 4F.tif]

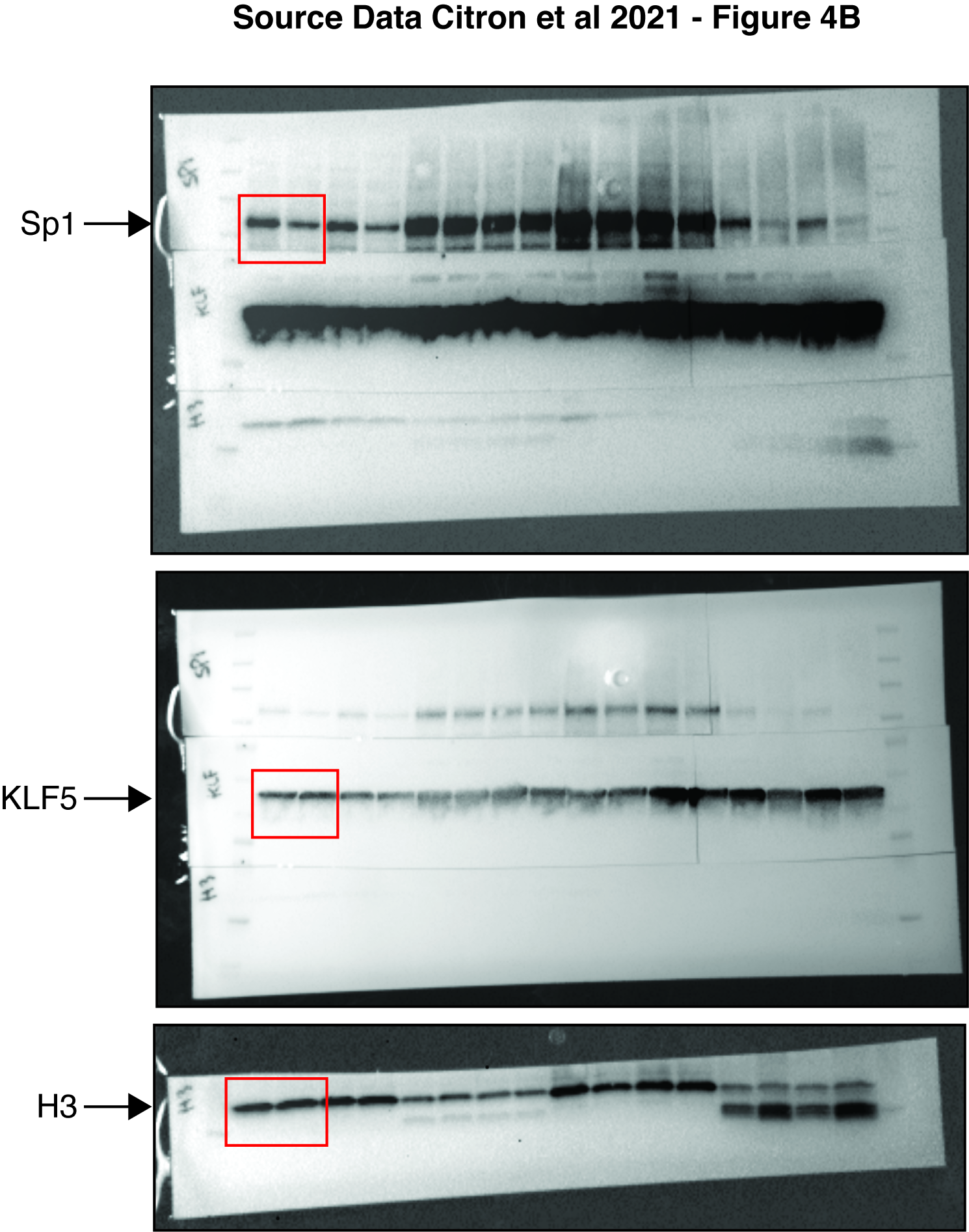

Supplement: Supplementary file 6 — Source Data for Figure 4 [file EMMM-13-e12872-s007.zip › Raw Data Fig 4B.tif]

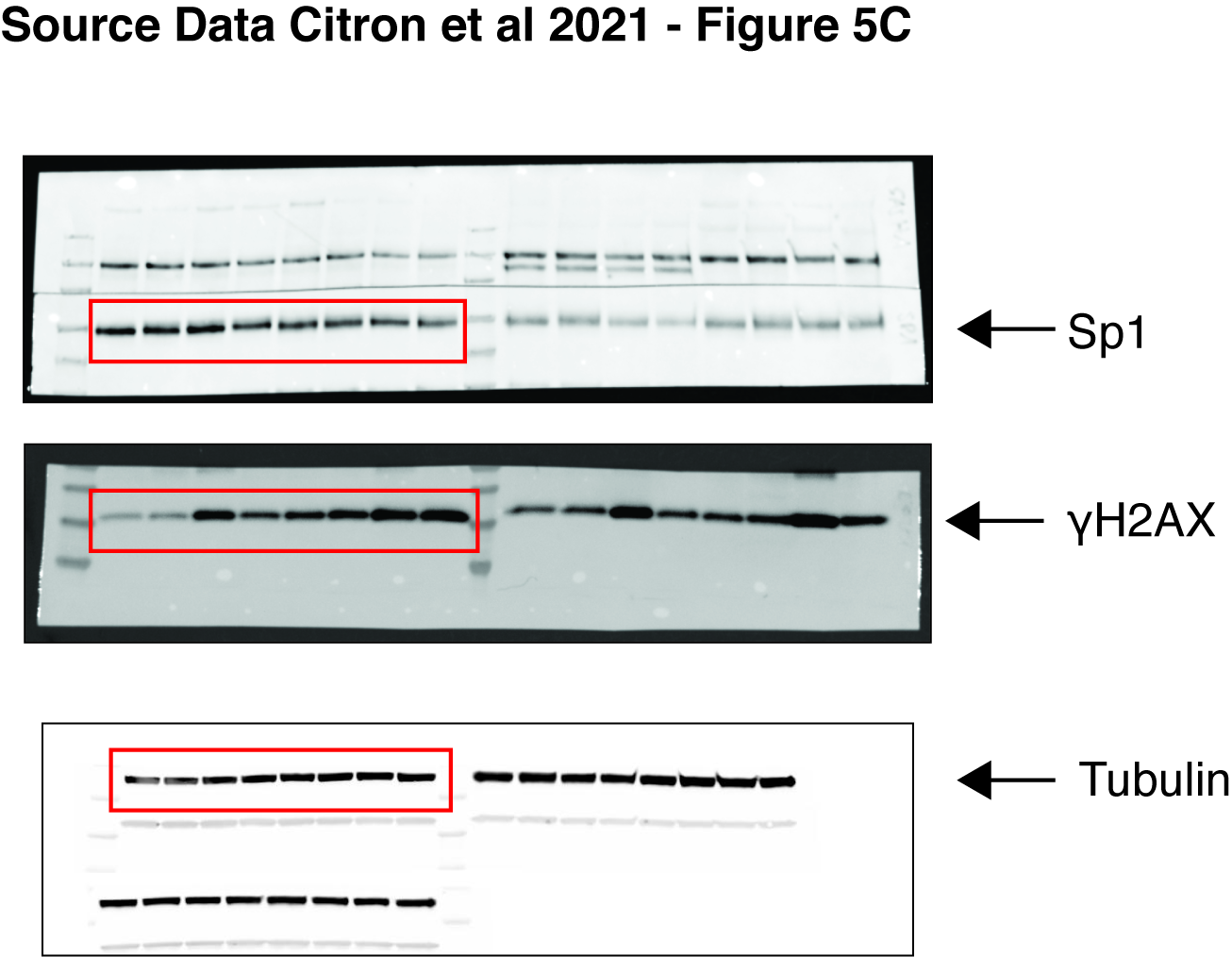

Supplement: Supplementary file 7 — Source Data for Figure 5C [file EMMM-13-e12872-s004.tif]
